# Assessment of changes of regional ventilation distribution in the lung tissue depending on the driving pressure applied during high frequency jet ventilation

**DOI:** 10.1186/s12871-018-0552-2

**Published:** 2018-07-31

**Authors:** Szymon Bialka, Maja Copik, Katarzyna Rybczyk, Aleksander Owczarek, Ewa Jedrusik, Damian Czyzewski, Marek Filipowski, Eva Rivas, Kurt Ruetzler, Lukasz Szarpak, Hanna Misiolek

**Affiliations:** 1 Chair and Department of Anesthesiology, Intensive Therapy and Emergency Medicine, Medical University of Silesia, Katowice, Poland; 2 Department of Statistics, School of Pharmacy with the Division of Laboratory Medicine in Sosnowiec, Medical University of Silesia, Katowice, Poland; 3 Chair and Department of Chest Surgery, Medical University of Silesia, Katowice, Poland; 4 Department of Outcomes Research, Cleveland Clinic, Cleveland, OH USA; 5 Hospital Clinic of Barcelona, IDIBPAS, University of Barcelona, Barcelona, Spain; 6 Lazarski University, 43 Swieradowska Str, 02-662 Warsaw, Poland

**Keywords:** Jet ventilation, Driving pressure, High frequency, Inspiratory pressure

## Abstract

**Background:**

Electrical impedance tomography (EIT) is a tool to monitor regional ventilation distribution in patient’s lungs under general anesthesia. The objective of this study was to assess the regional ventilation distribution using different driving pressures (DP) during high frequency jet ventilation (HFJV).

**Methods:**

Prospective, observational, cross-over study. Patients undergoing rigid bronchoscopy were ventilated HFJV with DP 1.5 and 2.5 atm. Hemodynamic and ventilation parameters, as well as ventilation in different regions of the lungs in percentage of total ventilation, assessed by EIT, were recorded.

**Results:**

Thirty-six patients scheduled for elective rigid bronchoscopy. The final analysis included thirty patients. There was no significant difference in systolic, diastolic and mean arterial blood pressure, heart rate, and peripheral saturation between the two groups. Peak inspiratory pressure, mean inspiratory pressure, tidal volume, and minute volume significantly increased in the second, compared to the first intervention group. Furthermore, there were no statistically significant differences between each time profiles in all ROI regions in EIT.

**Conclusions:**

In our study intraoperative EIT was an effective method of functional monitoring of the lungs during HFJV for rigid bronchoscopy procedure. Lower driving pressure was as effective in providing sufficient ventilation distribution through the lungs as the higher driving pressure but characterized by lower airway pressure.

**Trial registration:**

The study was registered on ClinicalTrials.gov under no. NCT02997072.

## Background

High frequency jet ventilation (HFJV) is a time cycled, pressure limited mode of ventilation that facilitate gas exchange by utilizing smaller tidal volumes from a high pressure at supraphysiologic frequencies between 120 and 400 min^− 1^ followed by a passive expiration [1]. HFJV offers the advantage of sufficient ventilation of patients lung’s and simultaneous low motion of the patients airway including the vocal cords. Consequently, HFJV is commonly used in Ear-Nose-Throat surgery, thoracic surgery and for rigid bronchoscopy [2, 3].

Mechanical ventilation with high tidal volume and low levels of positive end-expiratory pressure can promote ventilator induced lung injury (VILI), and thus increase morbidity and mortality [4]. Additionally, inhomogeneity in lung aeration exerts unequal stress on the lung parenchyma and might be another important contributor. Standardized measurements of ventilation parameters like tidal volume, peak volume or lung compliance, do not reliably translate the regional distribution of tidal volumes [5, 6].

Electrical Impedance Tomography (EIT) is a non-invasive, radiation free technology to measure the electrical impedance of the lungs, which corresponds to ventilation. In short, better ventilated parts of the lung are represented by higher electrical impedance. The impedance is measured via 16 surface electrodes attached via an elastic band around the patient’s chest and transmitted to the device. The metrics are processed to colorful images, allowing real-time continuous visualization and observation of regional differences of the ventilated lungs [7]. The EIT has been proven in various clinical settings to be a reliable and accurate bedside monitoring system of the ventilation [8–13].

Increasing driving pressure (Pplat – PEEP) in intubated and ventilated patients does have an impact on improving regional ventilation, but whether this improvement is also applicable in patients under HFJV is unknown [14, 15]. We tested the hypothesis, that increasing driving pressure from 1.5 to 2.5 atm caused increased regional ventilation distribution in 4 a priori defined regions of interests (ROI) in the lung.

## Methods

We aimed to investigate, if increasing driving pressure causes increased ventilation of dependent lung areas.

This is a prospective, crossover clinical study. With approval from the institutional review board of the Medical University of Silesia, Katowice, Poland (identifier: KNW/0022/KB1/42/16; Chairperson Prof M. Trusz-Gluza), and written informed consent, we enrolled 36 patients having elective rigid bronchoscopy requiring HJFV. The patients were enrolled at the Medical University of Silesia between July 2014 and December 2016, and the study was registered in clincialtrials.gov on (identifier: NCT02997072; posted on ClinicalTrials on December, 2016).

Participating patients were between 18 and 60 years, had an American Society of Anesthesiologists (ASA) physical status I-III, and a body mass index (BMI) of 19–30 kg/m^2^.

We excluded patients having emergency surgeries, known airway trauma and thoracic wall deformities, obesity (BMI > 30 kg/m^2^), and cardiac implantable electronic devices (pacemaker, ICD).

### Protocol

Patients were premedicated with oral midazolam 0.1 mg kg-1 one hour prior to arrival to operating room as clinical routine. After arrival in the operating room, the EIT electrode belt (*PulmoVista 500, Drager, Medical GmbH, Germany*) was placed around patient’s thorax under the 5th intercostal space. The reference electrode was placed on patient’s abdomen according to manufacturer’s instructions. Patients were also monitored with continuous 5 lead ECG, non-invasive blood pressure, and peripheral oxygen saturation as clinical routine.

General anesthesia was induced with propofol 2 mg/kg^− 1^, mivacurium 0.15 mg/kg^− 1^ and fentanyl 1.5 μg/kg^− 1^. Additional medication was given as clinically appropriate.

After confirmation of complete muscle relaxation by absence of palpable twitches in response to supra-maximal train-of-four stimulation of the ulnar nerve at the wrist, the rigid bronchoscope (Karl Storz, Germany) was introduced by the surgeon. Once optimal visualization of the vocal cords could be achieved, position of the bronchoscope was fixed, and the HFJV (*Universal Jet Ventilator Monsoon DeLuxe Acutronic, Switzerland*) was connected via the side channel of the bronchoscope. Initial ventilation setting included driving pressure of 1.5 atm, inspiratory time of 40%, gas humidifying 40%, and a respiration rate of 180/min (= first intervention group). The proximal end (visor) of the bronchoscope was always open during the complete surgery.

After 5 min of ventilation, the driving pressure was increased to 2.5 atm, while the other ventilation settings remained unchanged (=second intervention group). The fraction of oxygen was 1.0 throughout the complete procedure.

Peak Inspiratory Pressure (PIP), Mean Inspiratory Pressure (MIP), Tidal Volume (TV) and Minute Ventilation (MV) were monitored via bronchoscope side channel with jet catheter.

General anesthesia was maintained by continuous infusion of propofol and titrated as clinically appropriate. Continuous deep neuromuscular blockade was confirmed throughout the procedure and boluses of mivacurium were given per clinical judgement. After end to the surgical procedure, the rigid bronchoscope was removed and patients were intubated with a standard single lumen tube. Afterwards patients were transferred to the post-anesthesia care unit (PACU). Mechanical ventilation was maintained and muscle relaxation was reversed. Once patient fully recovered from anesthesia, patients were extubated and remained in the PACU as clinically appropriate.

### Measurements

To characterize distribution of regional ventilation, we a priori defined 4 regions of interest (ROI 1–4), which are based on the technical characteristics of the device used. The analyzed ROI divides the lungs into horizontal layers from dorsal-to ventral position. We decided to use this particular ROI, based on the assumption, that dependent lung areas are likely to develop atelectasis during general anesthesia. The use of EIT technology allows to determine the size and location of this atelectasis. During the study, the spread of ROIs were adjusted so that all layers were equal. Interpretation of the functional images relates to distribution of ventilation within the EIT sensitivity region and its changes over time. Numerical values indicate percentage of total ventilation in each region within 15 s time periods.

During an EIT examination, minimal alternating electrical currents were applied through pairs of electrodes and the resulting voltages are measured on the remaining electrodes. The most widespread spatial pattern of current applications and voltage measurements is through adjacent electrode pairs. Functional EIT images are generated from series of raw images and the corresponding pixel EIT waveforms, by the use of appropriate mathematical equations. Interpretation of the functional images allows to assess distribution of ventilation within the EIT sensitivity region and its changes over time. Numerical values indicate percentage of total ventilation in each layer within 15 s time periods.

### Statistical analysis

Statistical analysis was performed using STATISTICA 10.0 PL (StatSoft, Poland, Cracow). Statistical significance was set at a *p* value below 0.05. All tests were two-tailed. Interval data were expressed as mean value ± standard deviation. Distribution of variables was evaluated by the Shapiro-Wilk test and homogeneity of variances was assessed by the Levene test. For comparison of data between driving pressure 1.5 atm and driving pressure 2.5 atm groups, one-way ANOVA analysis with repeated measurement was done with contrast comparison as post-hoc tests. The distance-weighted least squares method was used to illustrate time changes of tidal volumes in four regions of interest separately for each driving pressure group.

## Results

**Fig. 1:**
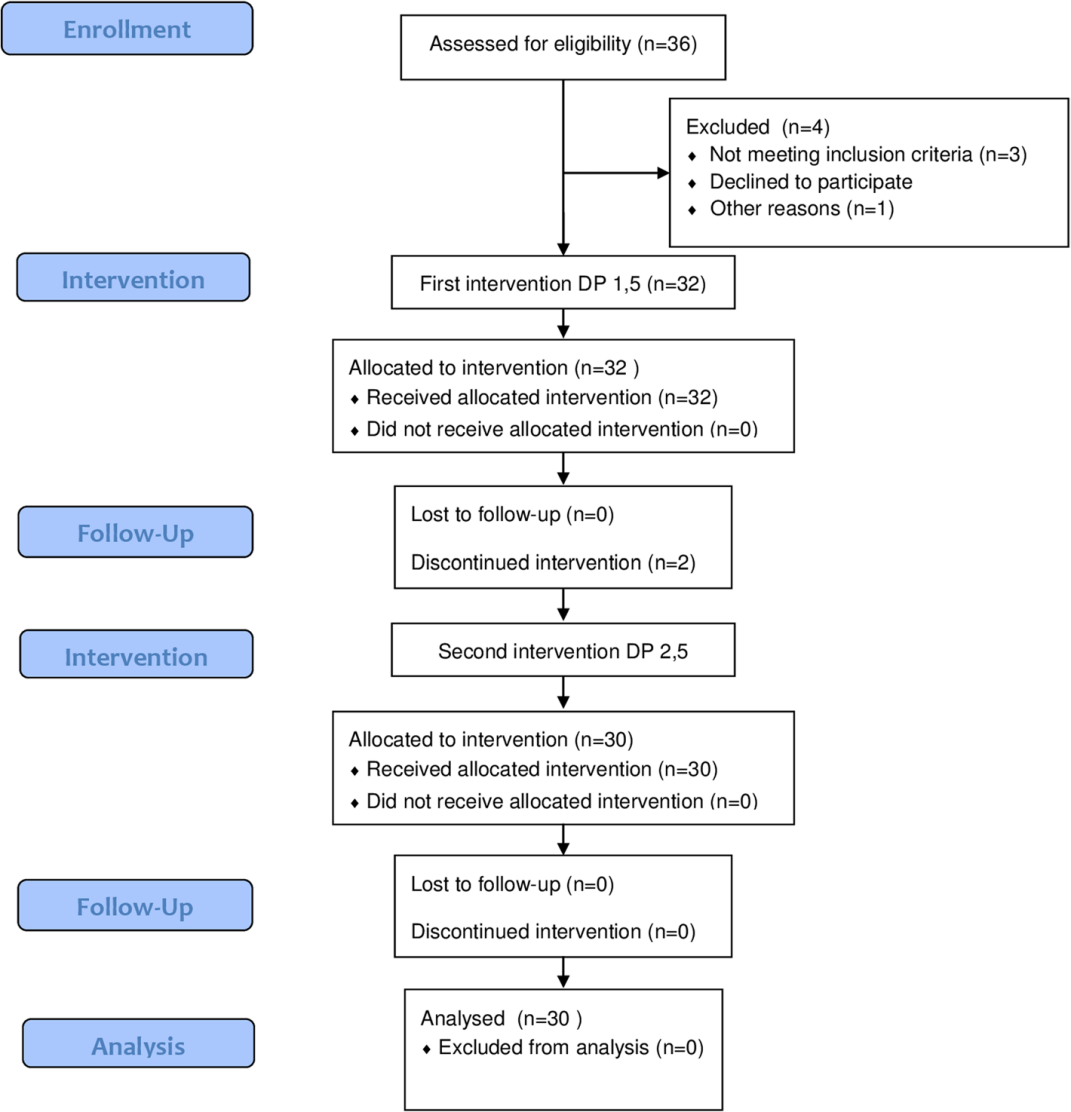
Flow diagram of the study

Thirty-six patients were enrolled into this clinical study, but four patients had to be excluded. The remaining 32 patients were included into this study and underwent the first intervention (driving pressure of 1.5 atm). Surgical procedure ended in 2 patients, before the second intervention was started. Consequently 30 patients (16 females, 61,5 years old and BMI 26,9 kg/m^2^), underwent also the second intervention (driving pressure of 2.5 atm) were included in the final analysis (Fig. 1).

**Fig. 2:**
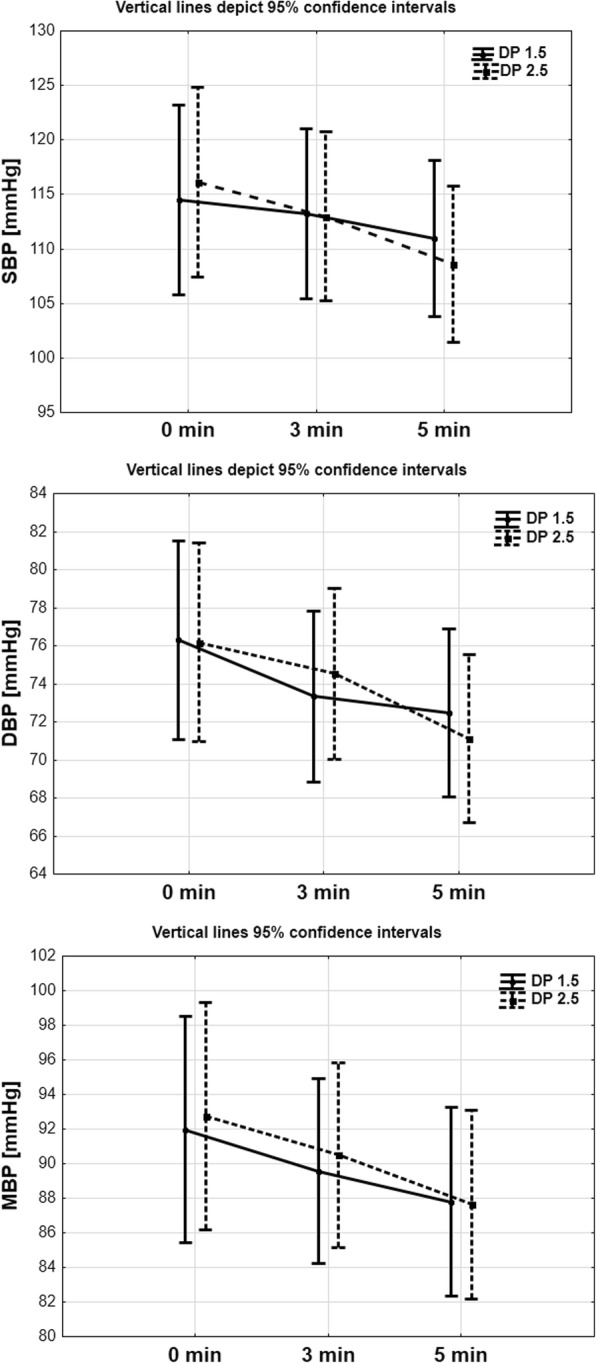
Comparison Systolic Arterial Pressure (SBP, mmHg), Diastolic Arterial Pressure (DAP, mmHg) and Mean Arterial Pressure (MAP, mmHg) in 5th minute observed time in DP 1,5 and DP 2, 5 group

The results of parametric analysis of variance with repeated measurements are presented in Table 1. There was no significant difference in systolic, diastolic and mean arterial blood pressure, heart rate, and peripheral saturation between the two groups (Fig. 2).

**Table 1:** Results of parametric analysis of variance with repeated measurements and descriptive statistics

	DP 1.5			DP 2.5			P_GROUP_	P_TIME_	P_INTERACTION_
	0 min	3 min	5 min	0 min	3 min	5 min			
SBP [mmHg]	114.5 ± 22.8	113.2 ± 18.9	110.9 ± 16.7	116.1 ± 24.8	113.0 ± 23.4	108.6 ± 22.2	0.95	**< 0.05**	0.55
DBP [mmHg]	76.3 ± 14.6	73.3 ± 10.8	72.5 ± 10.1	76.2 ± 14.0	74.5 ± 13.6	71.1 ± 13.8	0.97	**< 0.01**	0.60
MBP [mmHg]	92.0 ± 16.7	89.6 ± 14.0	87.8 ± 12.3	92.7 ± 19.1	90.5 ± 15.1	87.6 ± 17.2	0.89	**< 0.01**	0.92
HR [beats/min]	80.7 ± 13.0	79.3 ± 13.3	78.9 ± 12.9	80.9 ± 12.9	80.7 ± 13.6	79.5 ± 14.4	0.83	0.06	0.56
SpO_2_ [%]	97.5 ± 2.9	97.5 ± 3.2	96.9 ± 3.8	98.3 ± 1.7	98.0 ± 2.5	98.1 ± 2.1	0.23	0.20	0.18
PIP [cm H_2_O]	6.2 ± 2.8	6.3 ± 2.7	6.3 ± 2.7	9.4 ± 4.3	9.3 ± 4.2	9.5 ± 4.2	**< 0.01**	0.47	0.25
MAP [cm H_2_O]	2.5 ± 1.3	2.5 ± 1.3	2.4 ± 1.2	4.5 ± 2.2	4.4 ± 2.1	4.4 ± 2.1	**< 0.001**	0.28	0.75
TV [mL]	121.6 ± 6.8	121.5 ± 6.7	121.5 ± 6.8	149.2 ± 6.5	150.0 ± 5.3	149.8 ± 5.2	**< 0.001**	0.42	0.44
MV [L]	21.8 ± 1.2	21.7 ± 1.1	22.0 ± 1.5	26.5 ± 0.9	26.6 ± 0.6	26.6 ± 0.6	**< 0.001**	0.28	0.43

Interaction – between group and times

**Fig. 3:**
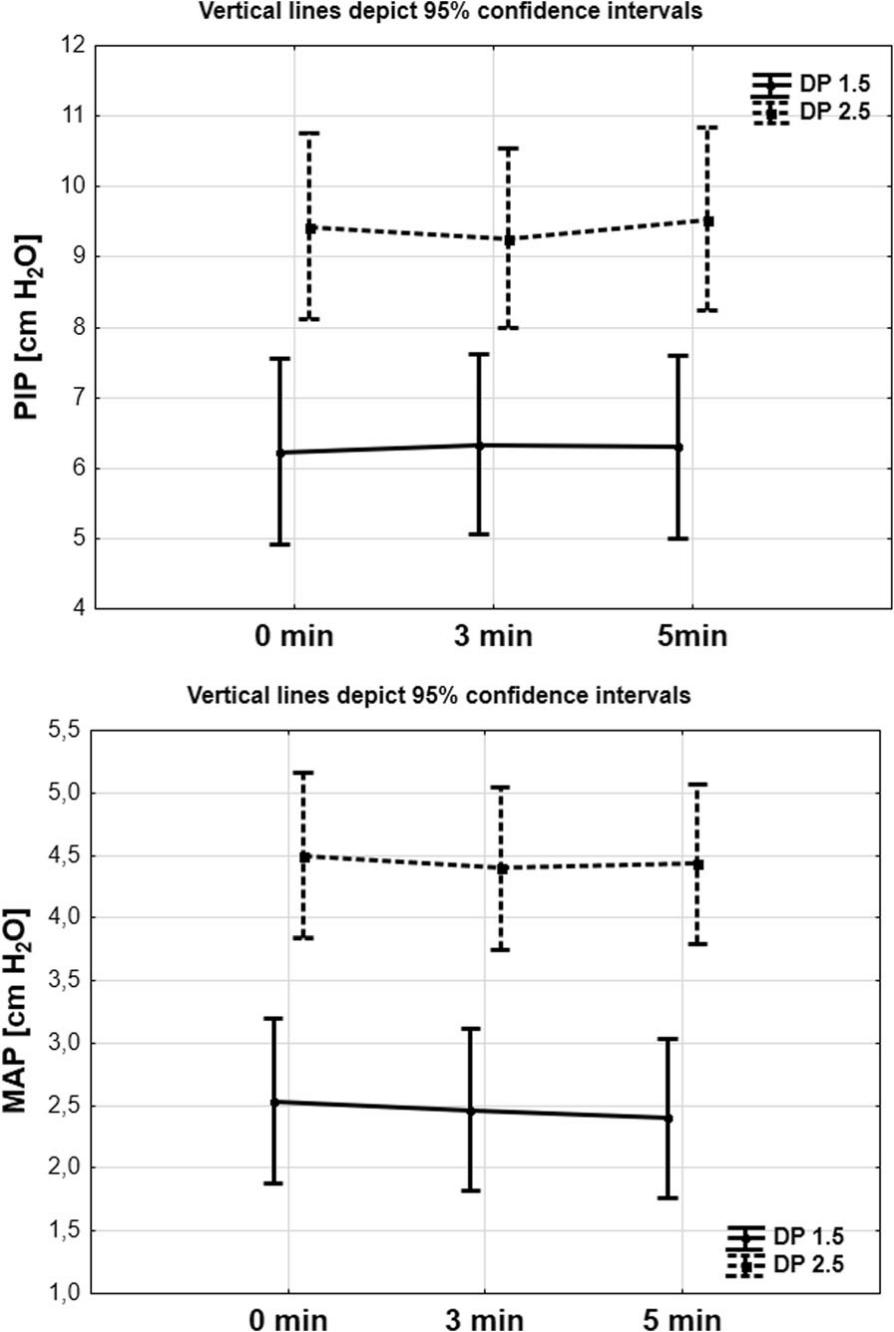
Comparison Peak Inspiratory Pressure (PIP, cm H2O) and Mean Inspiratory Pressure (MIP, cm H2O) in 5th minute observed time in DP 1, 5 and DP 2,5 group

Peak inspiratory pressure, mean inspiratory pressure, tidal volume, and minute volume significantly increased in the second, compared to the first intervention group (2.5 versus 1.5 atm; Table 1; Fig. 3).

**Fig. 4:**
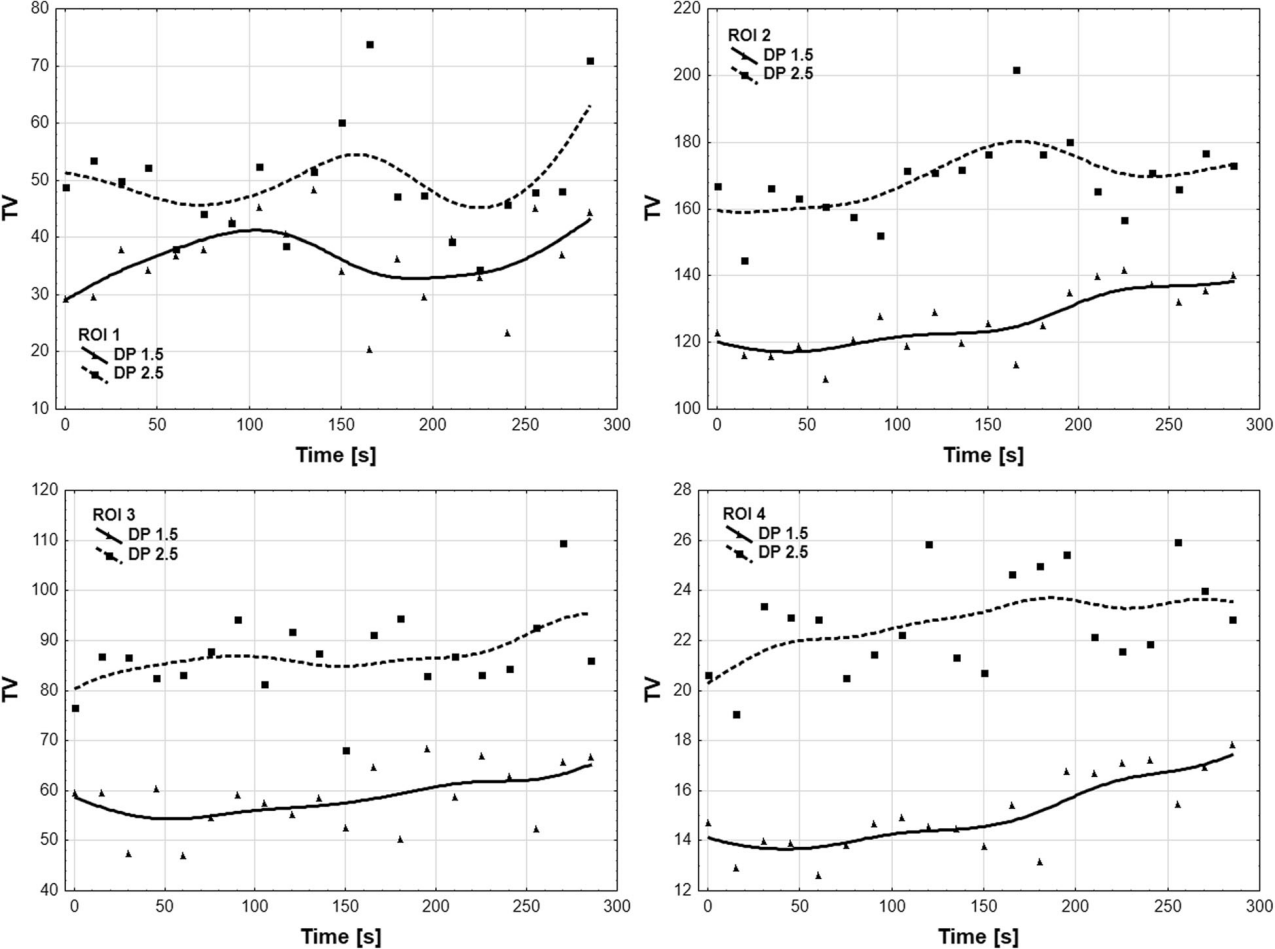
Comparison of Electrical Impedance Tomography (EIT) images in ROI 1 to ROI 4 in DP 1, 5 and DP 2,5 group

Tidal volume time changes between groups (DP 1.5 vs 2.5) were calculated. There were no statistically significant differences between each time profiles in all ROI regions:

- ROI 1: group influence (*p* = 0.73), group and time interaction (*p* = 0.29),
- ROI 2: group influence (*p* = 0.71), group and time interaction (*p* = 0.65),
- ROI 3: group influence (*p* = 0.41), group and time interaction (*p* = 0.94),
- ROI 4: group influence (*p* = 0.40), group and time interaction (*p* = 0.19; Fig. 4).

## Discussion

The most important finding of this study is, that increased driving pressure did increase overall tidal volumes, but this was mostly based on over-inflation of ventilated lung areas, instead of distributing ventilation to less ventilated lung areas.

The EIT was previously reported as an accurate method to detect ventilation inhomogeneity and was validated by several methods including radionuclide scanning, spirometer, and single-photo emission computed tomography [16–19]. Images gained by the EIT technology were previously used to identify the lung areas which are under and over ventilated (collapse or overinflated) [20].

During general anesthesia and in supine position, non-dependent parts of patient’s lungs are preferentially ventilated. Consequently, the lung strain is distributed heterogeneously among various regions of lung, with the maximum along the anteroposterior axis [21, 22]. This heterogeneity in distribution leads to atelectasis and potentially impairs CO_2_ elimination [23].

We decided to compare the driving pressure of 1.5 atm versus 2.5 atm and hypothesized, that increased driving pressure will improve regional ventilation distribution in dependent regions. Results of our study indicate, that ventilation in all lung areas increased, but non-dependent areas were still much better ventilation compared to dependent areas. Consequently, we did not find any hints, that increased tidal volumes are based on any significant shift of ventilation.

Increased driving pressure is applied in the clinical setting in order to provide higher tidal volumes, which is reflected by results of our study. Increased tidal volumes was mostly due increased ventilation of already ventilated non-dependent lung areas (represented as ROI 2 in our study). Dependent lung areas like the ROI 4 in our study were also more ventilated, but tidal volumes were still minimum. Increased tidal volumes and thereby caused stretch of lung tissues is potentially worrisome by causing local inflammation via interleukins (IL4, IL6) which is known to initiate ventilator induced lung injury [24]. Extra driving pressures across inhomogeneous lung may cause surfactant deactivation and alveolar edema disrupting alveolar interdependence leading to inhomogeneous ventilation [25].

Intraoperative ventilatory management is definitely not trivial, as this significantly impairs postoperative respiratory course [26]. Specifically increased driving pressure, is associated with increased risk of postoperative pulmonary complications [27]. Consequently, anesthesiologist should aim to reduce the driving pressure to a minimum level, as clinically appropriate.

The findings of pour study have some limitations. First, patients in our study tended to be slimmer compared to the general population. Higher BMI affects chest wall compliance due to the mass load effect and secondary causing increased pleural pressure. Airway driving pressures may vary over a wide range in patients according to the BMI. Second, we did not measure arterial oxygenation or CO_2_ elimination. Third, this study was designed as a sequential study and although physiologically highly unlikely, previous ventilation with lower driving pressure might have affected subsequent ventilation with higher driving pressure. However, a randomized ventilation strategy might have addressed this potential interference. Fourth, improved ventilation consequently leads to better oxygenation, which was outside of the scope of the current project and consequently, arterial blood analyses were not performed. Finally, although of being of clinical interest, we did not assess atelectasis after the procedure and any clinical complications like pneumothorax.

## Conclusions

Applying higher driving pressure is commonly used in the clinical setting to increase tidal volumes in order to maintain adequate oxygenation. Increased tidal volumes were achieved by over-inflation of already ventilated non-dependent lung areas, instead of any significant shift to less ventilated dependent lung areas.

## Abbreviations

ASA: American Society of Anesthesiologists
BMI: body mass index
DP: driving pressure
ECG: electrocardiogram
EIT: electrical impedance tomography
HFJV: high frequency jet ventilation
MIP: mean inspiratory pressure
MV: minute ventilation
PACU: post-anesthesia care unit
PIP: peak inspiratory pressure
TV: tidal volume
VILI: ventilator induced lung injury
